# Supplementary figures and images for: Catecholamines alter the intrinsic variability of cortical population activity and perception
Source: PLoS Biol. 2018 Feb 8;16(2):e2003453. doi: 10.1371/journal.pbio.2003453 (PMC5821404; doi:10.1371/journal.pbio.2003453)

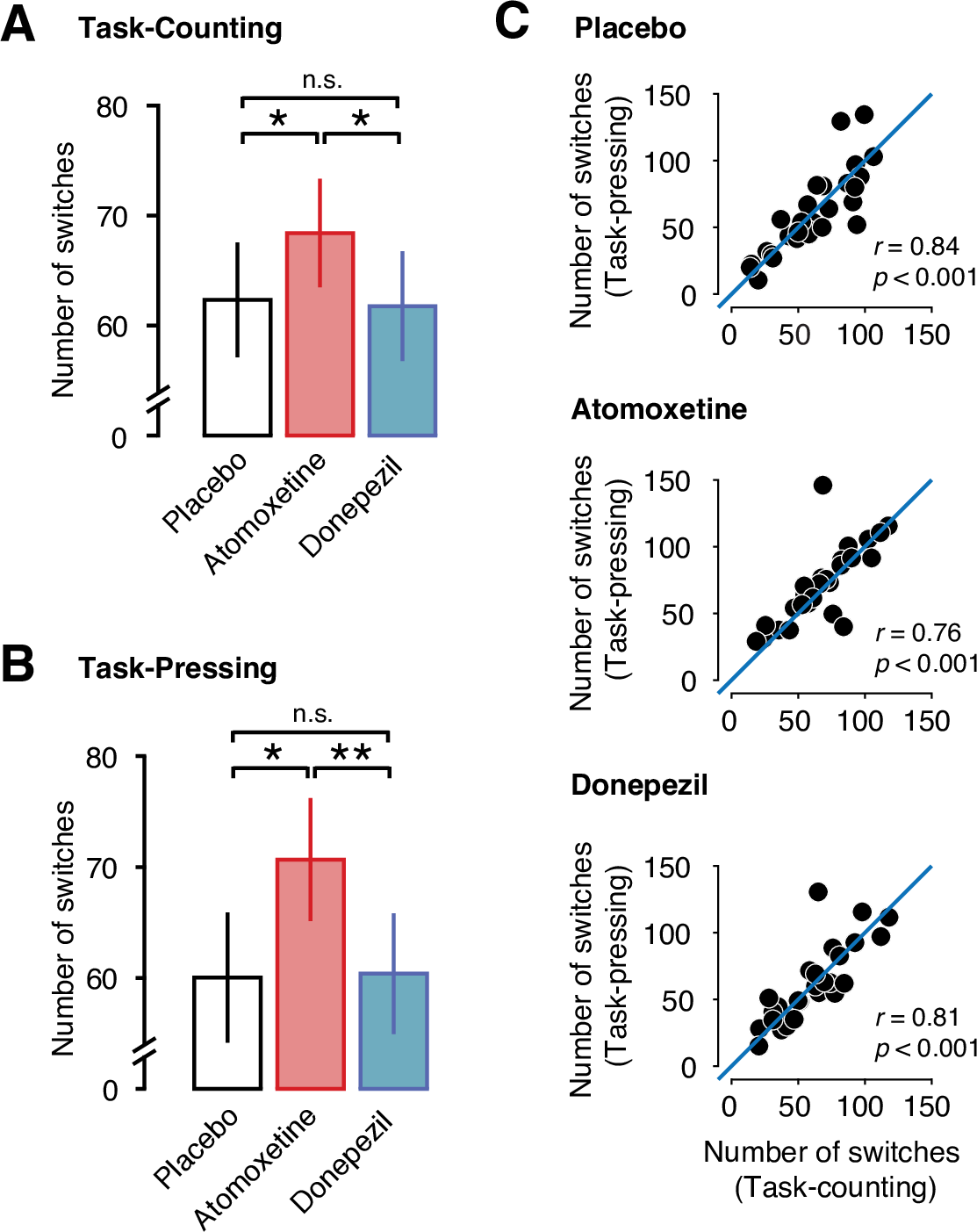

Supplement: S1 Fig — (A) Number of perceptual alternations reported by the subjects per 10-min run for the Task-counting condition. (B) Same as (A), but for Task-pressing condition. (C) Relation between the number of reported alternations during Task-counting (x-axis) and Task-pressing (y-axis). The blue line depicts a linear relation, with slope 1 as a reference. Two-sided t tests and Pearson correlations (N = 28). The data can be found at https://figshare.com/articles/Behavioral_data_Task-counting_/5756001. (TIF) [file pbio.2003453.s001.tif]

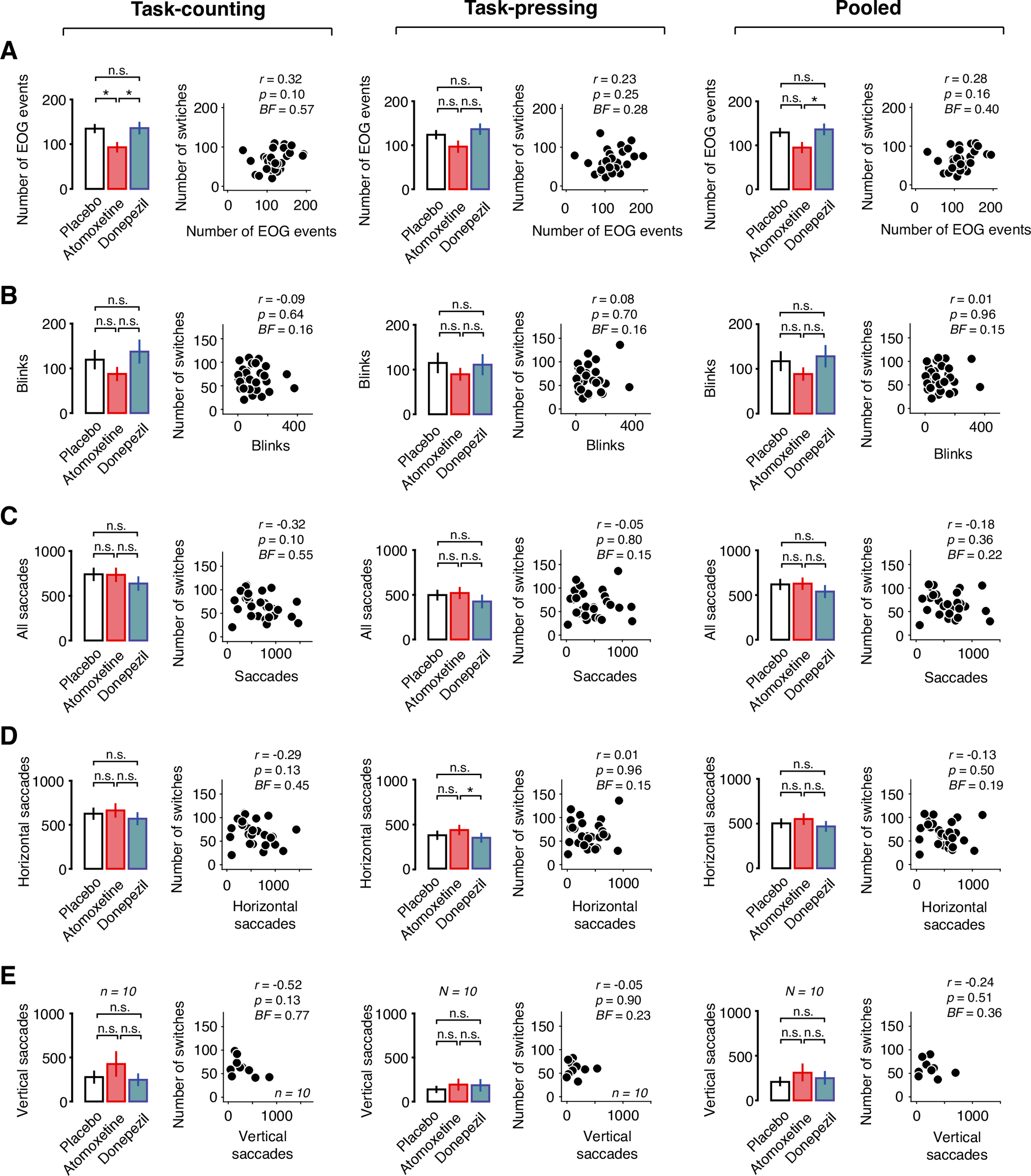

Supplement: S2 Fig — (A) Number of EOG events during Task-counting (left), Task-pressing (middle), and pooled across both conditions (right). Scatterplots depict the relation between the number of EOG events (x-axis) and the number of reported perceptual alternations (y-axis). (B) Same as (A), but for the number of detected eye blinks. (C) Same as (A) and (B), but for the number of saccades (horizontal and vertical). (D) Same as (C), but for horizontal saccades only. (E) Same as (D), but for vertical saccades only. Two-sided t tests and Pearson correlations (N = 28). These control analyses demonstrate that the change in perceptual dynamics under atomoxetine is not explained by changes in ocular parameters. The data can be found at https://figshare.com/s/a75b0d932c2e930f1d06. BF, Bayes factor; EOG, electrooculogram. (TIF) [file pbio.2003453.s002.tif]

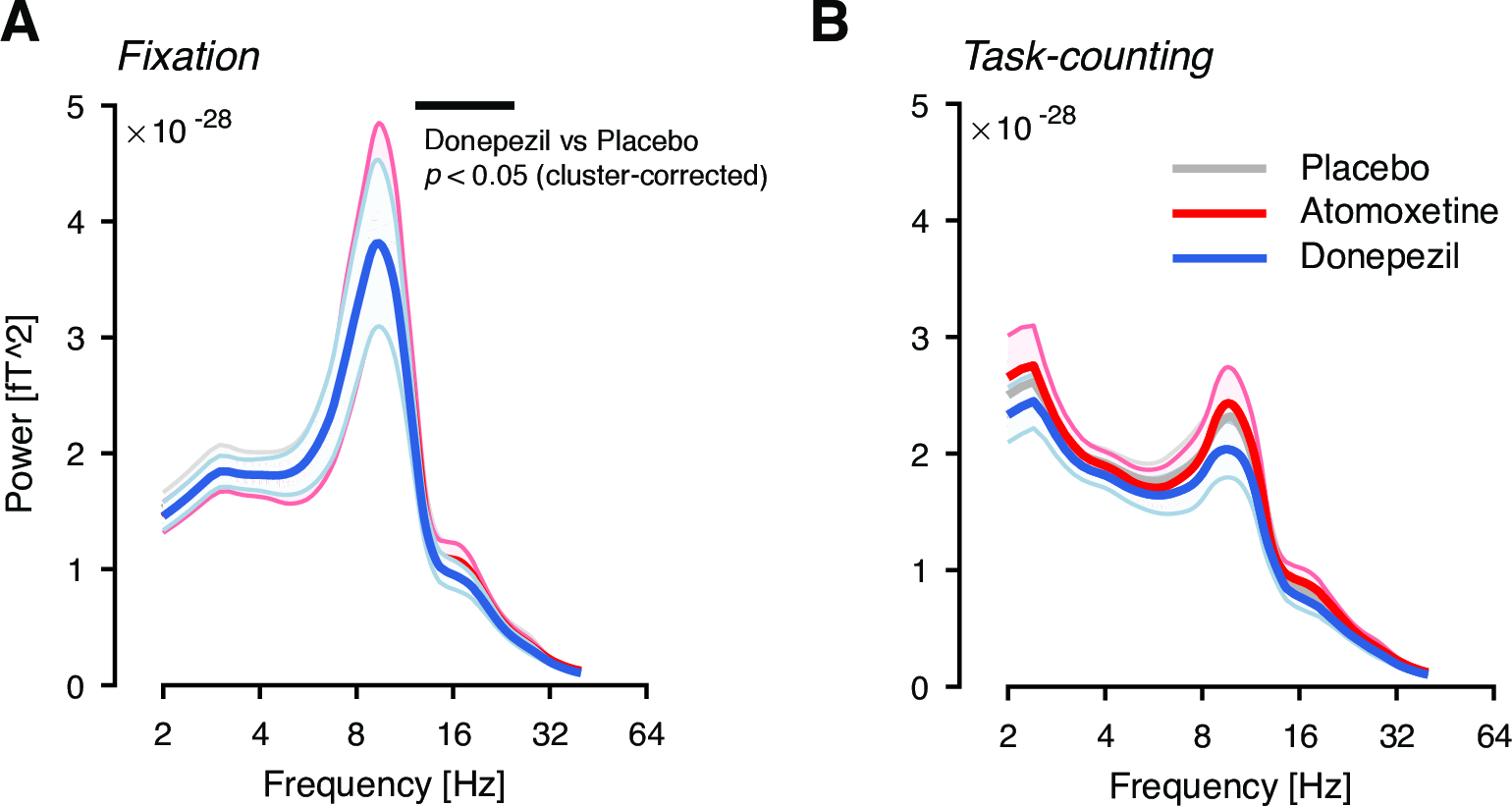

Supplement: S3 Fig — Black bar denotes significant differences assessed using a paired cluster-based permutation test (p < 0.05). The data can be found at https://figshare.com/s/ccefac78c698061219b5. MEG, magnetoencephalography. (TIF) [file pbio.2003453.s003.tif]

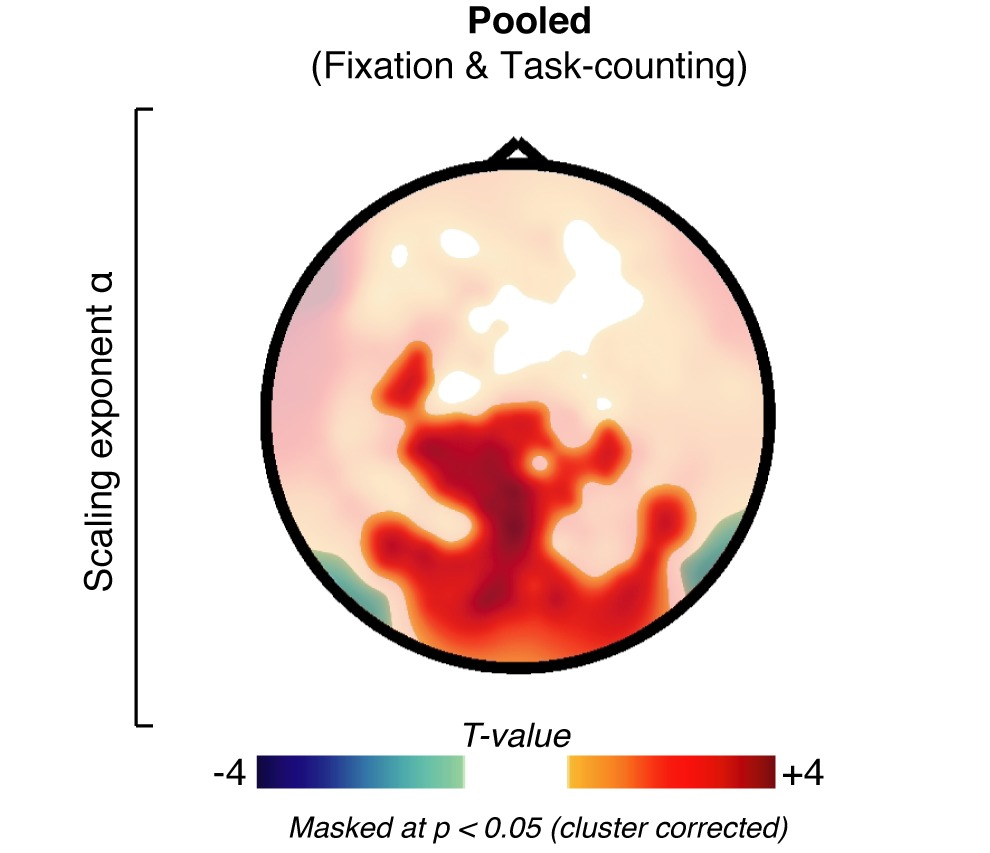

Supplement: S4 Fig — Thresholded at p = 0.05, two-sided cluster-based permutation test. The data can be found at https://figshare.com/s/34a7070a329772f90df8. (TIF) [file pbio.2003453.s004.tif]

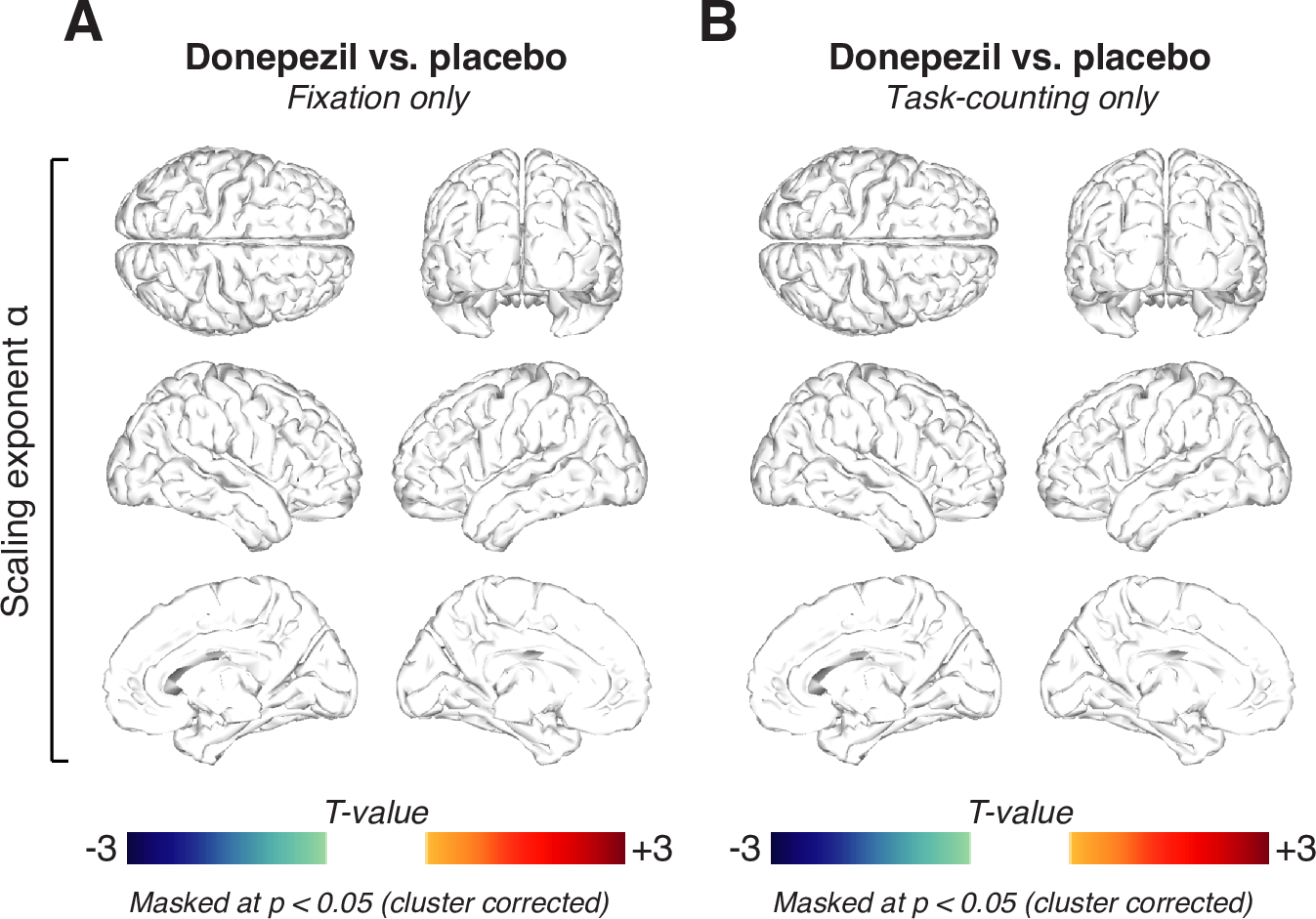

Supplement: S5 Fig — (A) Spatial distribution of Donepezil-induced changes in scaling exponent α during Fixation, thresholded at p = 0.05 (two-sided cluster-based permutation test). (B) As (A), but for Task-counting. The data can be found at https://figshare.com/articles/DFA_source_level_/5755311. (TIF) [file pbio.2003453.s005.tif]

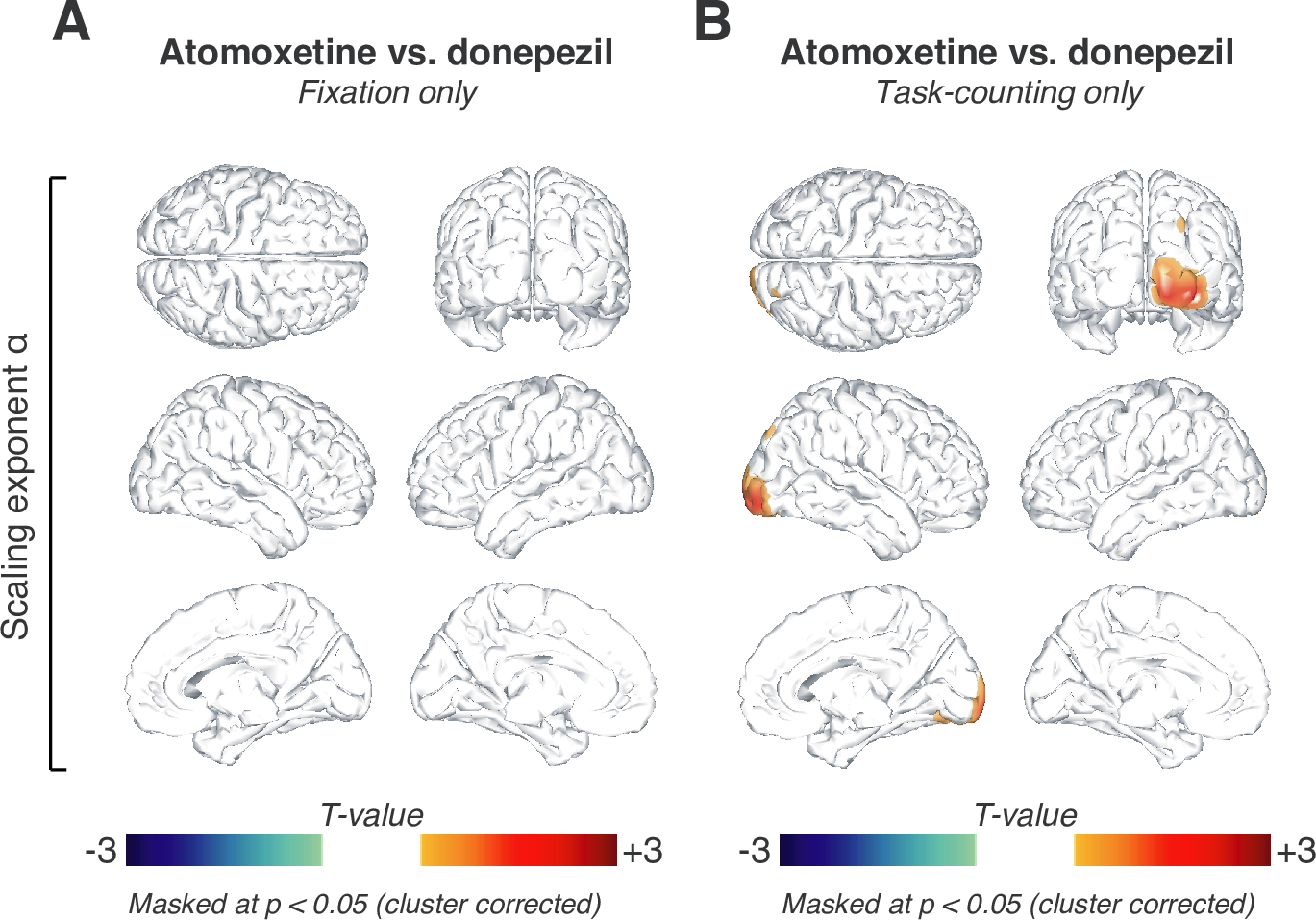

Supplement: S6 Fig — (A) Comparison of the effects of the two drugs conditions (i.e., Atomoxetine versus Donepezil) during Fixation. (B) Same as (A), but during Task-counting. All thresholds at p = 0.05, cluster-based two-sided permutation tests (N = 28). The data can be found at https://figshare.com/articles/DFA_source_level_/5755311. (TIF) [file pbio.2003453.s006.tif]

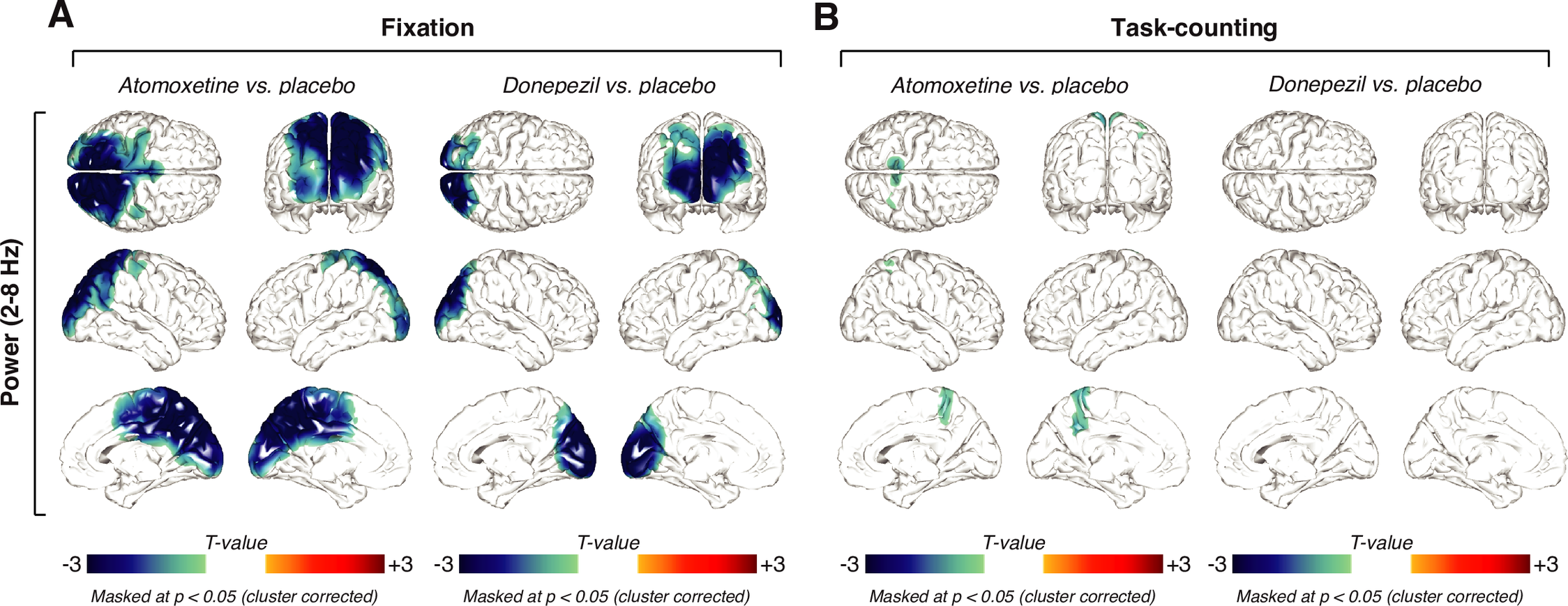

Supplement: S7 Fig — (A) Spatial distribution of drug-related low-frequency power changes during Fixation, thresholded at p = 0.05 (two-sided cluster-based permutation test). Left: Power changes after the administration of atomoxetine. Right: Power changes after the administration of donepezil. (B) Same as (A), but for Task-counting. The changes in low-frequency power in combination with the reported decreases in alpha-band power demonstrate a robust effect of both drugs on cortical dynamics. The data can be found at https://figshare.com/s/374ab0f973f026535549. (TIF) [file pbio.2003453.s007.tif]

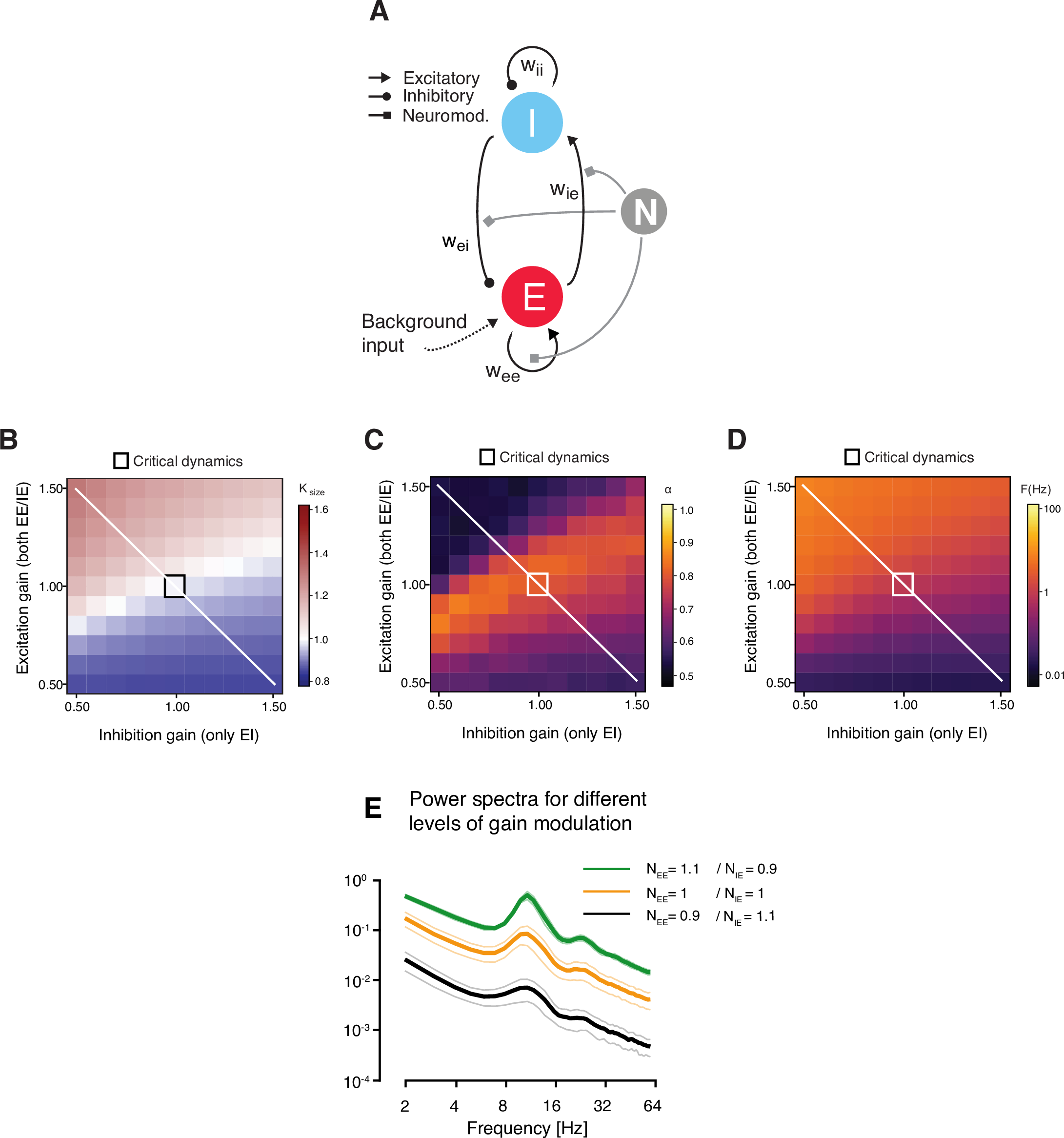

Supplement: S8 Fig — Different version of modulation of excitation-inhibition ratio in cortical patch model (A). Neuromodulation was simulated as a gain modulation term multiplied with excitatory (EE and IE) and/or inhibitory (EI only) synaptic weights. (B) κ as a function of excitatory and inhibitory connectivity (with a spacing of 2.5%; means across 10 simulations per cell). The region of κ~1 overlaps with the region of α > 0.5 and splits the phase space into an excitation-dominant (κ > 1) and an inhibition-dominant region (κ < 1). (C) Same as (B), but for scaling exponent α. (D) Same as (B) and (C), but for firing rate. In sum, the alternative version of modulation of excitation-inhibition ratio yields comparable results to the version presented in Fig 8. (E) Model power spectra under different levels of synaptic gain modulation (neuromodulation). The code and the data underlying these plots can be found at https://figshare.com/s/374ab0f973f026535549. EE, excitatory-to-excitatory (recurrent excitation); EI, inhibitory-to-excitatory; IE, excitatory-to-inhibitory; II, inhibitory-to-inhibitory (recurrent inhibition); κ, kappa index. (TIF) [file pbio.2003453.s008.tif]
